# DarkSky Halos: Use-Based Exploration of Dark Matter Formation Data in a Hybrid Immersive Virtual Environment

**DOI:** 10.3389/frobt.2019.00011

**Published:** 2019-03-04

**Authors:** Peter Hanula, Kamil Piekutowski, Julieta Aguilera, G. E. Marai

**Affiliations:** ^1^Electronic Visualization Laboratory, Computer Science Department, University of Illinois at Chicago, Chicago, IL, United States; ^2^Art Design and Architecture Department, University of Plymouth, Plymouth, United Kingdom

**Keywords:** immersive analytics, virtual reality environments, hybrid environments, astronomy, data visualization

## Abstract

Hybrid virtual reality environments allow analysts to choose how much of the screen real estate they want to use for Virtual Reality (VR) immersion, and how much they want to use for displaying different types of 2D data. We present the use-based design and evaluation of an immersive visual analytics application for cosmological data that uses such a 2D/3D hybrid environment. The applications is a first-in-kind immersive instantiation of the Activity-Centered-Design theoretical paradigm, as well as a first documented immersive instantiation of a details-first paradigm based on scientific workflow theory. Based on a rigorous analysis of the user activities and on a details-first paradigm, the application was designed to allow multiple domain experts to interactively analyze visual representations of spatial (3D) and nonspatial (2D) cosmology data pertaining to dark matter formation. These hybrid data are represented at multiple spatiotemporal scales as time-aligned merger trees, pixel-based heatmaps, GPU-accelerated point clouds and geometric primitives, which can further be animated according to simulation data and played back for analysis. We have demonstrated this multi-scale application to several groups of lay users and domain experts, as well as to two senior domain experts from the Adler Planetarium, who have significant experience in immersive environments. Their collective feedback shows that this hybrid, immersive application can assist researchers in the interactive visual analysis of large-scale cosmological simulation data while overcoming navigation limitations of desktop visualizations.

## 1. Introduction

The Electronic Visualization Laboratory (EVL) at the University of Illinois at Chicago has been at the forefront of Virtual Reality (VR) research since 1992, when it created the first CAVE environment, a projection-based virtual reality system. Since then, the laboratory has developed a wide range of immersive systems, including a second generation hybrid VR environment called CAVE2 in 2012 (Febretti et al., 2013). The CAVE2 is a passive hybrid reality environment that can show both 3D immersive representations and 2D representations using a system of large high-resolution tiled LCD displays. These technologies can be used for Immersive Analytics tasks—deriving insights from data by augmenting the human analysts' ability to make sense of the large and multifaceted datasets which are common across scientific disciplines.

In one example of such a large, multifaceted dataset facilitated by advancements in high-performance computing, cosmologists are able to model the formation of the universe via n-body simulation, from the Big Bang to the present. These simulations make up a core part of our understanding of the known universe. The volume of data generated by these simulations is enormous, with the largest simulation to date (Skillman et al., 2014) having produced data on the order of petascale. The task of analyzing and disseminating this level of data has, unsurprisingly, proved to be a major challenge. The need for visual analysis tools which can accurately represent the complex interactions of these simulations has only grown. Major challenges involved in addressing this visual analysis problem include scalability, access to distributed data sources, integration of spatial and non-spatial data, the ability to relate spatial structures to each other in space and over time, and even data processing procedures such as ensuring that derived spatial data points are correctly aligned.

**Figure 1:**
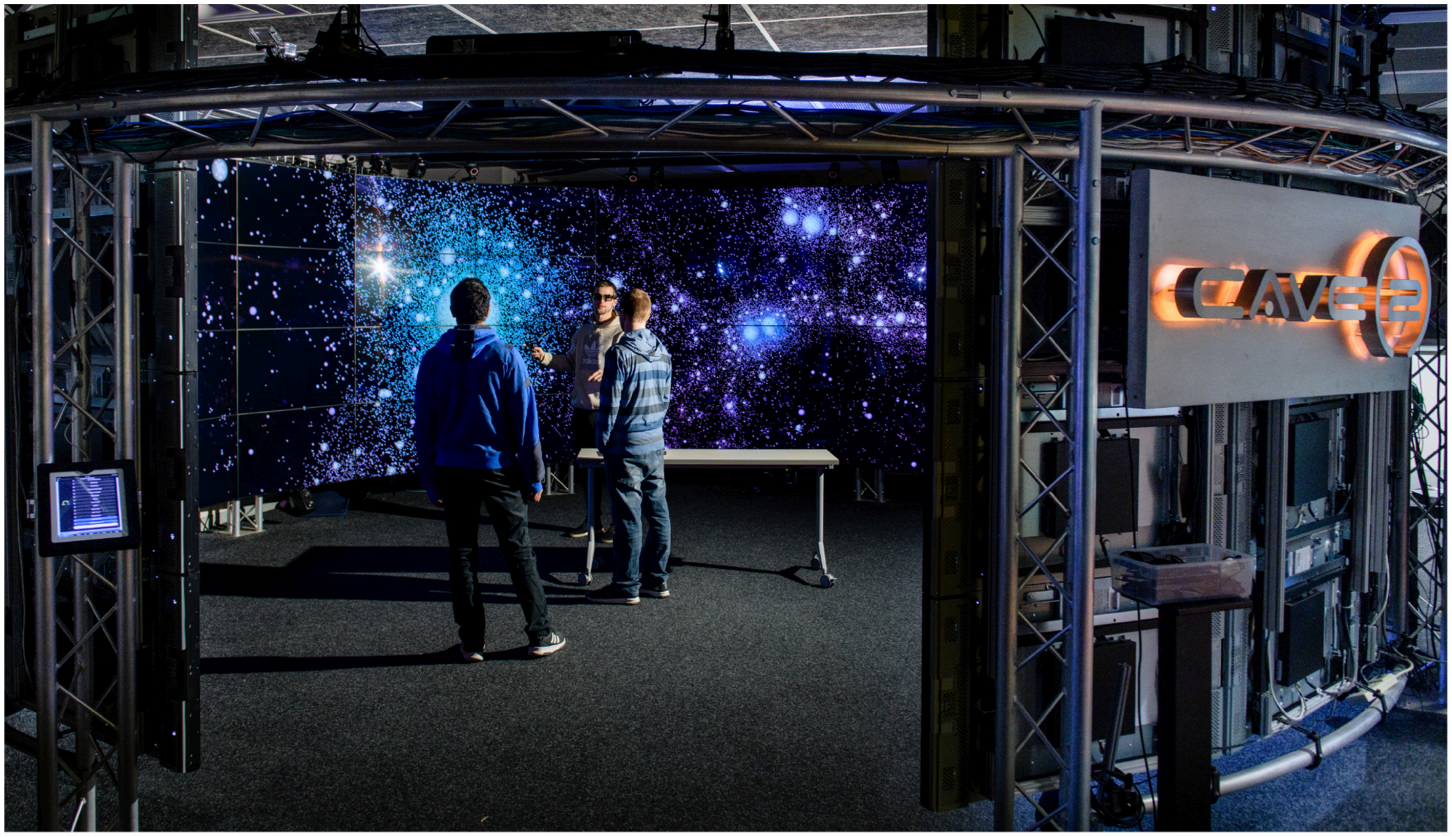
Collaboratively examining in an immersive environment the formation of dark matter halos.

In this paper we describe the use-based design and evaluation of an immersive visual analytics application for cosmological data that uses the CAVE2 hybrid environment (Figure 1). In a departure from human-centered design approaches, the design and evaluation are based on the theoretical framework of activity-centered visualization design. The design further follows a details-first paradigm based on scientific workflow theory. Last but not least, based on a rigorous analysis of the analyst activities, the application was designed to allow multiple domain experts to interactively analyze visual representations of nonspatial (2D) and immersive spatial (3D) cosmology data pertaining to dark matter formation.

## 2. Related Work

Thanks to increasing advancements of high performance computing, cosmologists are able to model the formation of the universe. Multiple attempts have been made to use visual analysis as a tool for exploration and analysis of several complex, large scale datasets in astronomy. Previous works have visualized, for example, the Millennium Simulation Project's 10 billion dark matter model of the expansion of the universe. Due to the size of the Millennium dataset, some of these approaches utilize snapshots of the data or pre-rendered animations (Springel et al., 2005). Other works have utilized multi-GPU mesh deformation to represent gravitational forces (Kaehler et al., 2012). Continuous level of detail of point clouds have also been used to interactively explore billions of particles (Fraedrich et al., 2009). Luciani et al. (2014) have tackled the visual analysis of astronomy images. Last but not least, a few works have attempted to visualize dark matter data, as we do (Almryde and Forbes, 2015; Christoudias et al., 2015; Hazarika et al., 2015). However, none of these works have explored the potential of immersive visual analytics in the context of astronomy data.

Many works in visualization design theory for visual analysis, most notably Tory and Möller (2004) and Sedlmair et al. (2012), investigate human-centered design in the context of domain-expert driven visualization problems. In contrast, comparatively little is known about the visual design process in the context of the activity-centered design paradigm (Marai, 2018) adopted in this work. In terms of the second theoretical paradigm instantiated in this work—details-first design—Chen et al. (2016) were the first to note, in 2016, that in “many scenarios, we often observe that an experienced viewer may find (overview first and details on demand) frustrating, as the viewer knows exactly where the interesting part of a detailed representation is. For example, in flow simulation, scientists work on the same problem for months.” While the theoretical work of Luciani et al. (2019) provides a scientific workflow framework for this paradigm, no immersive applications document its application in practice.

Furthermore, from the virtual reality end, there is surprisingly little work in the area of hybrid 2D-3D visual analytics. Work in our lab has previously explored 2D and 3D representations in a virtual reality environment in the context of a NASA project. The NASA-funded ENDURANCE team explored ice-covered Lake Bonney in the McMurdo Dry Valleys of Antarctica, and used our hybrid environment to explore simultaneously a 3D reconstruction of the lake in one half of the space, and a 2D bathymetric representation of the lake in the other half. The dual representations enabled the team to successfully capitalize on the complementary expertise of the group members. The space helped keep the team productive (Reda et al., 2015).

In this paper we present a novel approach toward the visualization of large-scale, n-body cosmological simulations of Dark Matter Particle and Halo data. The approach applies for the first time an Activity-Centered design paradigm to an immersive environment. The approach further follows, and documents for the first time in an immersive environment, a theoretical details-first design paradigm. We further incorporate an immersive 2D/3D hybrid display to facilitate the interactive exploration by domain experts of both spatial and nonspatial cosmological data. While preliminary results from this project have been previously submitted in the form of an abstract to the IEEE Scientific Visualization Contest (Hanula et al., 2015), no part of this work has been presented at a conference beyond the level of a poster presentation.

## 3. CAVE2 Hybrid Environment

The 2012 CAVE2 is a 74 megapixel 2D/37 megapixel passive 3D hybrid reality environment. The environment was designed and built based on lessons our laboratory learned from building the original projector-based CAVE in 1991, and from building the large high-resolution tiled LCD displays in the 2000s (Marai et al., 2016). Thirty-six computers drive 72 LCD panels (18 columns of 4) arranged in a 22' wide 320° circle. Fourteen Vicon tracking cameras allow the tracking of six items in the space (glasses and/or controllers) and a 20.2 surround system provides audio feedback. CAVE2 provides a space where groups of analysts have sufficient screen real estate and resolution to show multiple representations at the same time, from tables of numbers to fully immersive 3D spaces.

While the CAVE2 provides the hardware for an Immersive Analytics environment, the environment software uses SAGE2 (Renambot et al., 2016) and OmegaLib (Febretti et al., 2014). OmegaLib is the open source software we have developed to drive CAVE2 and other devices in fully immersive interactive mode. SAGE2, built primarily in JavaScript and HTML5, allows us to run 36 interlinked web browsers in the CAVE2 as one single shared canvas where multiple analysts can interact simultaneously, adding content to the walls, moving, resizing, and interacting with that content, and sharing their desktops. Analysts interacting with the immersive 3D world can use a tracked controller, while other members of the team are simultaneously interacting through their laptops or tablets. Running both SAGE and OmegaLib simultaneously allows the analysts to choose how much of the CAVE2 screen real estate they want to use for VR immersion, and how much they want to use for sharing different types of related 2D representations.

## 4. Domain Problem and Activity Centered Design

### 4.1. Domain Problem: Halo Evolution

Following a challenge from the IEEE VIS 2015 conference, an interdisciplinary team of EVL researchers set out to develop a visual analysis tool for large-scale cosmological simulations of dark matter formation (Hanula et al., 2016). The team consisted of visualization researchers and astronomy researchers. The data were provided by the Dark Sky project hosted by Stanford University. While the largest simulations (half a Petabyte) from this project contain 1.1 trillion particles and stretch 38 billion light-years across, the astronomers selected for analysis, and provided us with, a data subset (11 GB) that contains 2 million particles stretching 203 million light years across (Contest, 2015). Subsets of particles (10–100 K) from these 2 million particles merge into dark matter halos.

The Dark Sky project aims to model via simulations the process of dark matter structure formation in the Universe. The simulations interpret dark matter as a collisionless fluid, represented as a discretized set of particles that interact only gravitationally. Each simulation requires a large number of particles—typically on the scale of 10–100 K particles—to be simulated over 14 billion years. Over this time span, the particles form, via gravitational pull, structures that draw in and collapse the matter that forms stars, galaxies, and clusters of galaxies.

In the domain scientist workflow, dark matter structures are identified through a process known as halo finding, where halos represent either galaxies or clusters of galaxies. Dark matter halos are identified either via local particle density estimation or through simple linking-length mechanisms. Within a galaxy cluster, smaller halos (substructures) may be identified. As these structures and substructures interact, merge, separate, and grow, the domain scientists believe that the structure of the Universe grows and changes.

### 4.2. Activity-Centered Design

We approached the challenge by following an Activity-Centered-Design (ACD) paradigm for visualization (Marai, 2018), which is an extension of the classic Human Centered Design paradigm in visualization design. The approach places particular emphasis on the intended use of an analysis tool, through the close identification of analyst workflows. We adopted this approach because of its documented higher rate of success in interdisciplinary project settings. Because there are no other documented instantiations of the ACD paradigm in immersive environment applications, we describe this process in detail below.

We implemented the theoretical ACD paradigm through an iterative, multi-stage process. As described in detail below, this process included reviewing documents from a functional activity perspective, i.e., trying to identify the main expert activities to be performed as part of the visual analysis process (further described below). The activity-design process instantiation further included semi-structured interviews with a domain expert, a senior researcher at the Adler Planetarium, to confirm functional requirements related to the activities to be supported by the system, and to explore prototypes. The research team met then weekly with the domain expert over the course of 9 weeks, as the application was being developed and the design refined, to collect feedback and to verify that evolving requirements were being satisfied. We observed the domain expert's activity as the expert interacted with the application. Because of the exploratory visualization nature of the project, and in concordance with activity-centered design, we furthermore used a quantitative methodology to assess the capabilities of the application, and qualitative evaluation methodology to analyze the user activities on a homogeneous sample of participants who share key characteristics, described in detail in section 5.

Following the ACD theoretical stages for domain characterization (Marai, 2018), we examined first the activities and data behind the project. In the first stage of the ACD approach, we reviewed the challenge documents to identify the main activities to be performed by an analyst in the target domain. It became soon clear from the challenge documents that these activities were centered around the features of interest in the application, namely “halos.” Halos are sets of gravitationally bound particles that together form a coherent structure. Unfortunately, halos are difficult to identify computationally, due to the lack of a clear, predefined mathematical formula. The domain scientists were particularly interested in the interactive identification and visualization of halos. Second, they were interested in the analysis of halos' evolution over time: halos can appear, merge into larger coherent structures, split, and/or dissipate over time. Once halos and their temporal evolution were identified and explored, the properties of these coherent structures were also of interest. In particular, the particle distribution within a structure was of interest, as well as summaries of these particle distributions within a halo, such as angular momentum or the relative concentration of particles.

In terms of data analysis, the project involved three primary types of data. The first data type is the raw particle data, described by a position vector, a velocity vector, and a unique particle identifier. The second type of data is one Halo Catalog for each timestep in the simulation, for a total of 100 timesteps. The role of each catalog is to group together into coherent structures sets of gravitationally-bound particles. Along with information about a given halo's position, shape, and size, the catalog contains a number of statistics derived from the particle distribution, such as angular momentum and relative concentration of the particles within that halo. The final data type links the individual halo catalogs, thereby creating a Merger Tree database. These merger trees form a sparse graph that can then be analyzed to better understand how galaxies form and evolve through cosmic time. In terms of immersive analytics, it was clear that the spatial structure of the halos and their trajectories over space and time were central to the investigation. It became further clear that the scale of the data was going to pose clutter, obstruction, and navigation problems (helping the analysts not get lost in the data as they would zoom in) in non-immersive environments, making the problem an excellent fit with immersive analytics.

Once we identified the main analyst workflow and the data used in this project, and further following the ACD theoretical model, we verified our understanding of the problem with a senior researcher at the Adler Planetarium in Chicago. The astronomy expert confirmed the result of our analysis. The expert's confirmation of the activity analysis concluded our implementation of the ACD domain characterization step.

The ACD theory further bears influence on the visualization design stage of the project. By focusing on the user activities, we noted that halo detection was essential to the expert workflow. We further noted the lack of a precise mathematical formula for identifying the halo features: the catalogs are derived on the basis of expert-tweaked thresholds. Given these observations, we considered the design problem from the perspective of scientific workflow theory and of the three known top-design visualization paradigms: “overview-first” (Shneiderman, 1996), “search-first” (van Ham and Perer, 2009), and “details-first,” later formally documented in the visualization literature as a theoretical model (Luciani et al., 2019). Following the workflow activities, together with the domain expert we agreed on an overall design that emphasized the features of interest first, then provided spatial and temporal context information, and finally provided a summarization overview of the data (Luciani et al., 2019). This overall design implements in an immersive environment the details-first paradigm: details-first, show context, overview last. We describe in detail our implementation of this paradigm.

### 4.3. Visual Encodings and Implementation

Our resulting design consists of an immersive linked multiview display which allows domain experts to interact with visual representations of spatial and nonspatial cosmology data. We used the CAVE2 immersive environment, the D3 API and the OmegaLib framework for virtual reality to display the “detail” 3D particles and halos, as well as the context temporal data. Spatial data was represented through GPU-accelerated point clouds and geometric primitives. The D3 nonspatial views were displayed into the immersive environment using D3 and SAGE2. Last, the nonspatial data was represented as time-aligned merger trees, and an overview was provided via a pixel-based heatmap. Interaction and a communication channel between D3 and OmegaLib allowed spatial and nonspatial views to be linked. Spatial-nonspatial integration through linked views has been previously shown to effectively bridge, as a visual scaffold, complementary representations of data (Marai, 2015).

**Figure 2:**
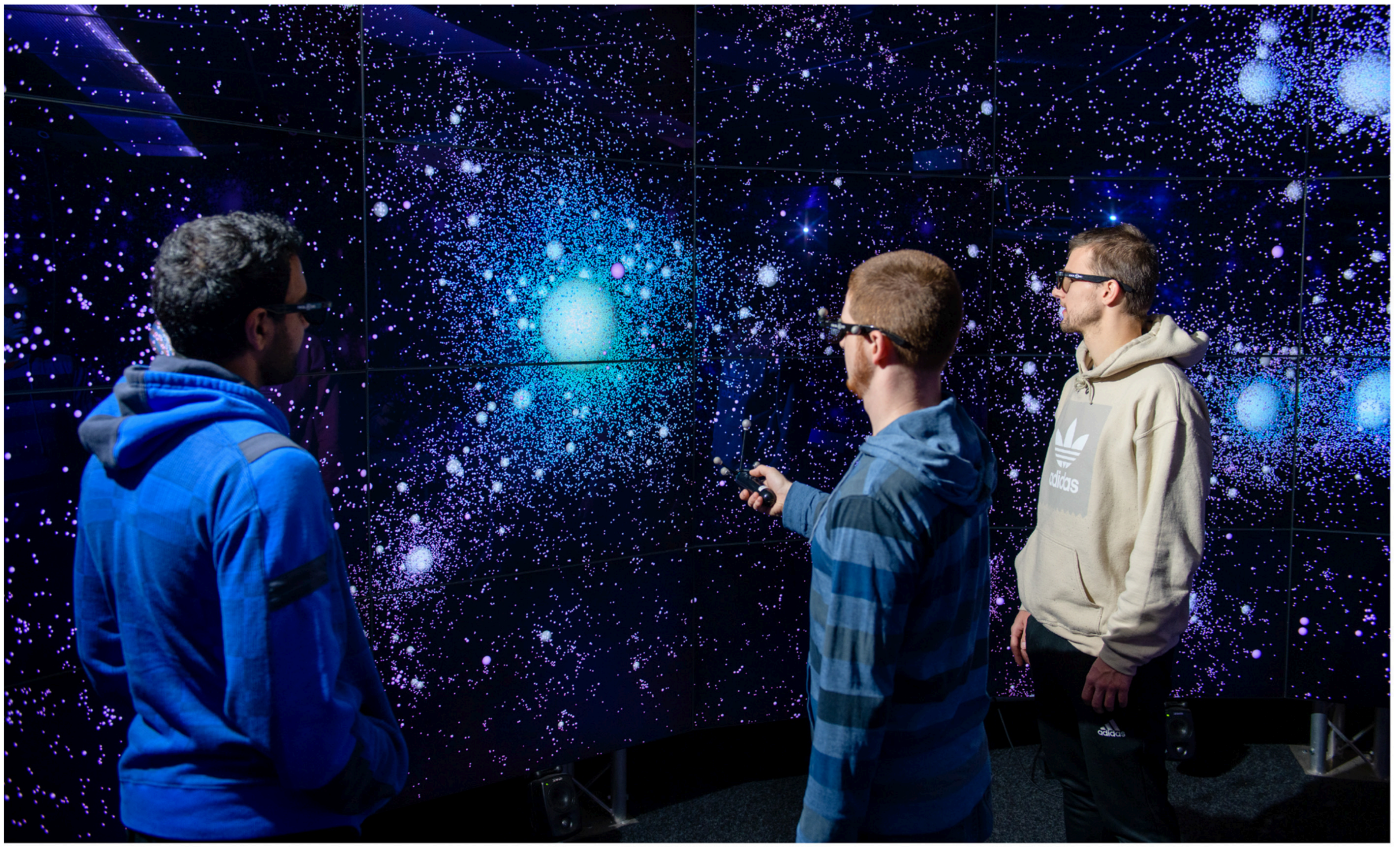
Single timestep view of several large halos which are starting to cluster. Note the halo structures, along with the substructure particles that surround the halos. Halos are a transparent white, while substructure particles are colored by potential. Each tile of the immersive display is the size of a regular desktop display, indicating how little information and context could fit on a single display.

**Figure 3:**
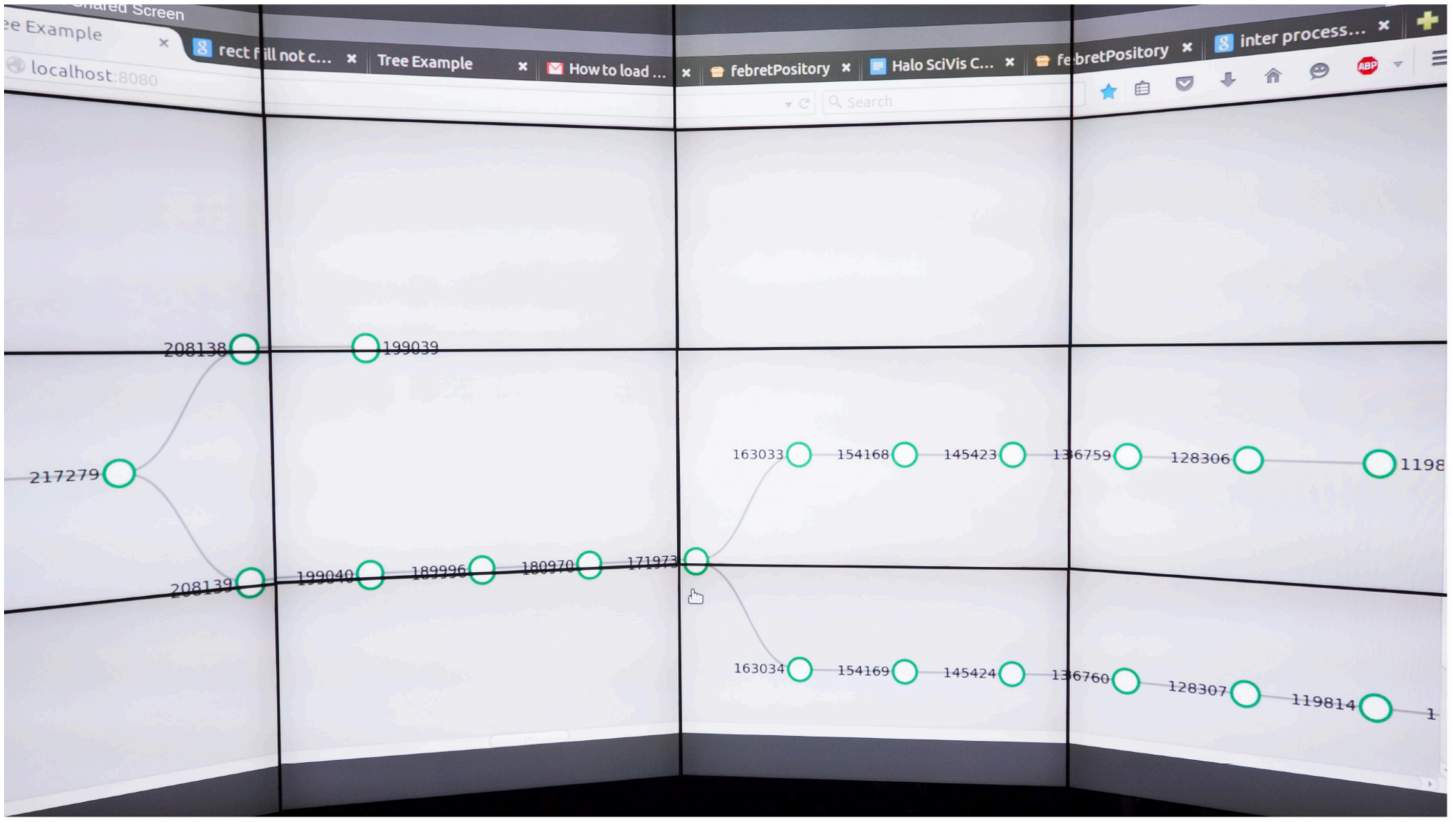
2D representation of a Merge Tree, with time mapped to the horizontal axis. In the tree representation, nodes represent halos, and the arcs represent merging and splitting operations. Note how halos merge, get created, or disappear through time.

The entry point of the application is the details-first immersive exploration of a particular timestep or time lapse interval (Figure 2). An analyst may browse the corresponding 3D particle set and halos, then navigate to the merger tree data. A second view shows a 2D representation of the selected tree, with time mapped on the horizontal axis (Figure 3). From here, the analyst can select a particular time step, or a time lapse interval, and immersively explore, in the immersive view, the corresponding 3D particle set and halos.

**Figure 4:**
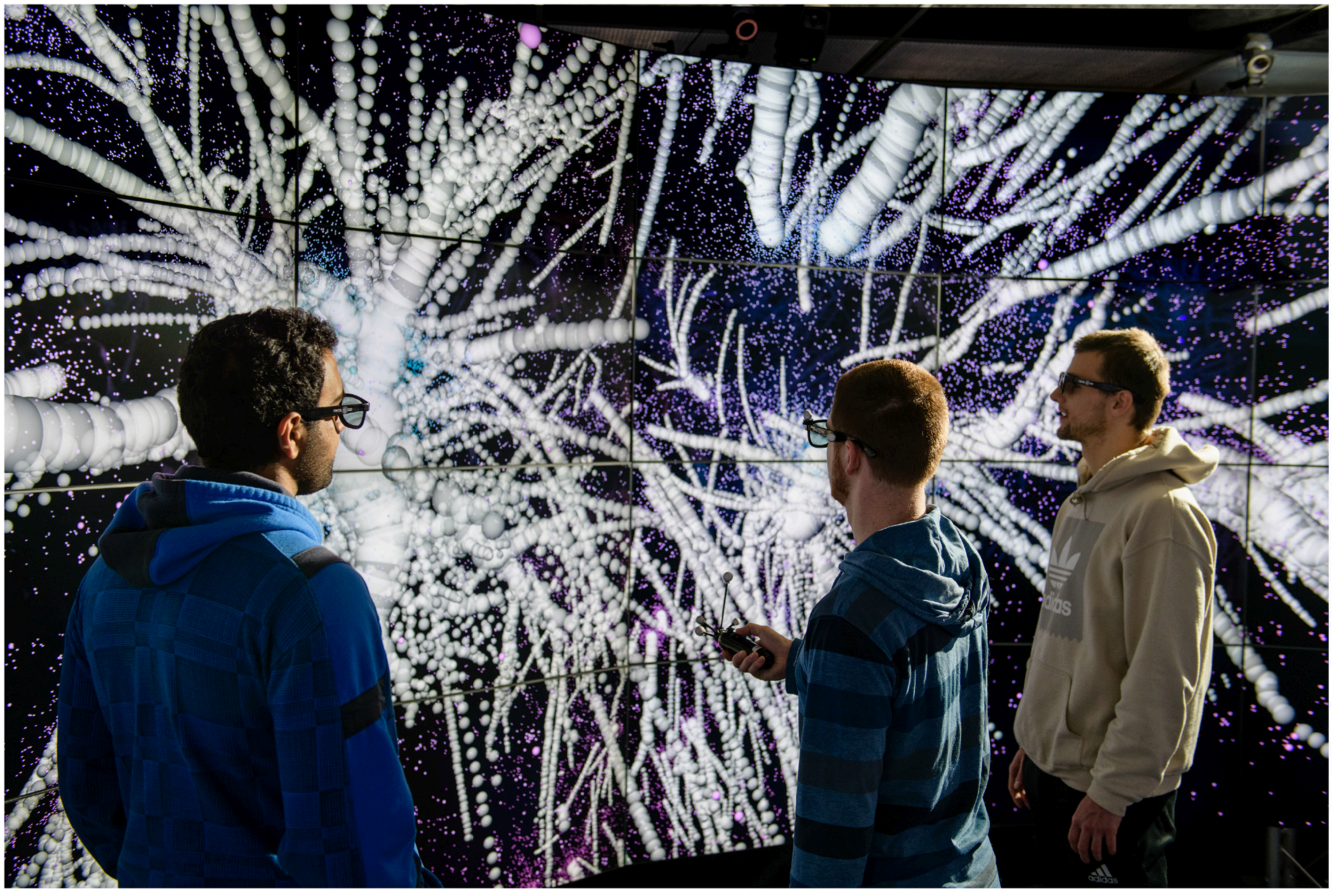
Time lapse detail of 89 timesteps, which shows the paths taken over time by halos. Note how halos merge, get created, or disappear through time. With 3D glasses, we can see depth of the halos and the paths they form, as well as where halo formations start and eventually end. Each tile of the immersive display is the size of a regular desktop display, indicating how little information and context could fit on a single display.

**Figure 5:**
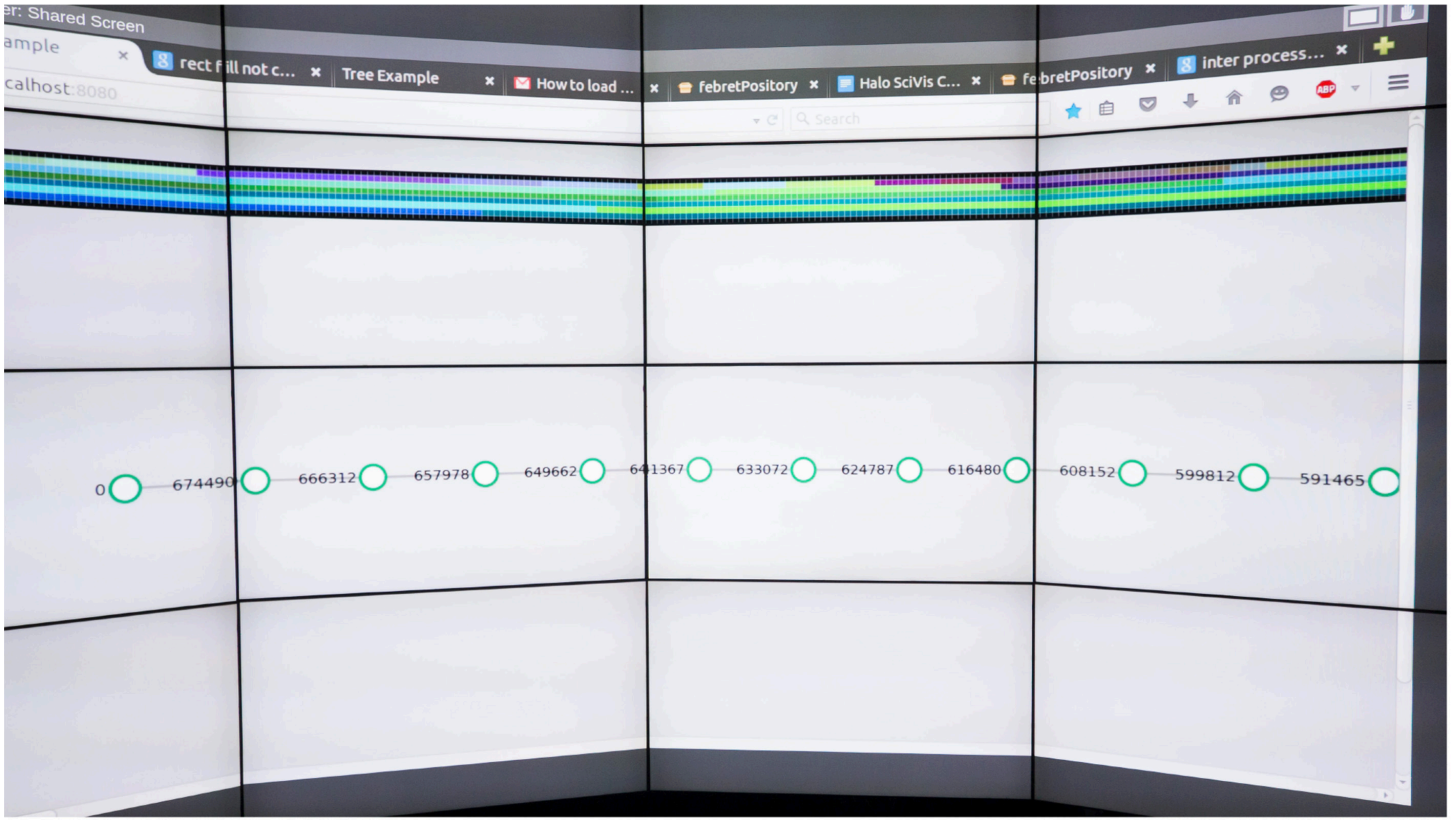
Pixel heatmap (top) and time-aligned tree representation (below). Each heatmap square represents a tree in the tree forest. The heatmap intensities can be mapped to specific characteristics of each tree—for example, the tree depth, breadth, the size or mass of the largest halo in the tree, or the end timestamp of each tree. From this summarization overview of the tree data, the analyst can select a particular tree of interest.

We further implemented a 3D time lapse function (Figure 4), which can overlap several selected timesteps to show the flow and path of the halos and/or particles over time. The time lapse creates a static 3D representation of the merger trees. The representation can also be animated to show the halo formations at each timestep. While the animation is playing, the analyst can freely move through the environment and zoom in on a desired halo formation. The time lapse visualization can be used to show all the merger-trees in 3D space. Finally, a pixel heatmap shows a summarization overview of the merger tree forest, i.e., the collection of all merger trees in the simulation data (Figure 5). The heatmap intensities can be mapped to specific characteristics of each tree—for example, the tree depth, breadth, the size, or mass of the largest halo in the tree, or the end timestamp of each tree. From this summarization overview of the tree data, the analyst can select a particular tree of interest.

Below we describe in detail our data pre-processing steps and each visual encoding.

#### 4.3.1. Data Ingesting

For developing initial visualization methods and interfaces, we used the 11G 128^3^ particle dataset provided through the IEEE SciVis 2015 website. In the pre-processing stage, we ran a python (van Rossum and Drake, 2001) script to parse the raw particle and halo catalog datasets, using yt (Turk et al., 2011), and thingking (Turk et al., 2014), respectively. After integration and processing (described below), the spatial particle and halo data are displayed and manipulated in our immersive environment using OmegaLib.

To read merger tree data we wrote a custom parser. To enable better data flexibility, we also wrote an algorithm to create a proper JSON format (ECMA International, 2013). In our approach, the branches expand backwards with respect to time: the algorithm starts at the final stage of the halo creation and moves back in time by recursively searching for the parents of each node.

#### 4.3.2. Data Integration and Processing

The particle data and the halo data are specified in the original datasets in different, specific coordinates, and need to be cross-registered before displaying. As required by the challenge, for the particle dataset we used a modified formula to convert the proper *kpc* coordinates to *Mpc*/*h* comoving coordinates, as follows:

(1) $$\begin{array}{l} pos\_cMpc=\left(pos_{kpc}+width/2.0\right)*h_{100}*kpc\_to\_Mpc/cosmo_{a}\\ \;+redShift*Delta \end{array}$$

where *pos*_*kpc*_ represents the position coordinate to convert, *width* represents the simulation width, *h*_100_ represents the Hubble parameter, and *kpc*_*to*_*Mpc* represents the conversion rate. In turn, *cosmo*_*a*_ represents the scale factor for a particular snapshot, *redShift* represents the redshift value for a particular snapshot, and *Delta* = −31.2715 is an offset constant specified in the sample dataset.

After reading in the initial particle and halo parameters (XYZ coordinates, acceleration, velocity, and potential for raw particle data; XYZ coordinates, mass, radius, and circular velocity for halo data), we converted the data to the format used by OmegaLib. The Point Cloud module of OmegaLib was used to load and handle point cloud data. We extended the module to accommodate the parsed parameters mentioned above, and the opacity field, alpha, was replaced with an identifier to differentiate raw particles from halos. In total, 178 files were generated (89 for the raw particle dataset + 89 for the halo dataset), giving two files per timestep ranging from the twelfth timestep to the hundredth. These files were then loaded into memory in order to be further processed by our python program.

To take full advantage of GPU acceleration, we further normalized particle parameters such as potential, acceleration, or velocity to a common range of [0:1,000,000]. For the Halo dataset we normalized the Halo Mass, Circular Velocity, and other parameters to a smaller common range of [0: 300].

#### 4.3.3. Particle and Halo Encoding

The particle and halo data are visually encoded as point clouds, respectively as transparent spheres. The sphere radius is mapped to Rvir (Halo radius), a parameter provided by the Halo catalog dataset. The particles are color-mapped by potential (an indicator of the power of a halo), while halos are colored a semi-transparent white. The white encoding was the result of experimentation and enables the halos to stand out. The halo transparency further allows analysts to examine the particle substructure of a halo. Both the particle data and the halo data can be toggled off or on.

Analysts can choose between three color schemes: (1) cyan to purple particles with yellow halos, allowing the analysis to focus on general flow from a min to max data set for particles, with halos shown in clear contrast, (2) gray-scale particles with red halos, allowing a focus on halos and on the particles that make up the halo, or (3) multi-hue sequential colored particles with white halos, which makes it easier to distinguish the different ranges of data sets, and easier to examine the substructure of a halo. The third color scheme is the most clear and it is displayed by default.

The raw particle dataset can also be color-mapped by velocity and acceleration, while halos can be colored by any parameter specified by the analyst via an interactive menu. By default, all halos are the same color, making it easier to distinguish between particles and halos. However, when the particles are toggled off, the halo colors can be mapped to halo quantities of interest, for example, the halo mass.

#### 4.3.4. Time Lapse Encoding

In our implementation of a 3D time lapse function, the analyst can specify a start timestep, an end timestep, and the step size. The application overlaps the selected timesteps to show the flow and path of the halos and/or particles over time. The time lapse creates a static 3D representation of the merger trees. The representation can also be animated to show the halo formations at each timestep. While the animation is playing, the analyst can freely move through the environment and zoom in on a desired halo formation.

We use the OmegaLib's GLSL shaders to render points, and take thus advantage of GPU-acceleration when working with these large datasets.

#### 4.3.5. Time-Aligned Tree Encoding

The 3D tree time lapse representation above can give analysts a good sense of space and spatial relationships. However, the halo evolution is fundamentally a function of time, with different start and end timestamps for different halo trees. The sheer number of trees can also get quickly overwhelming in 3D. Last but not least, specific tree statistics are difficult to visualize and sift through in the time lapse view.

To circumvent these issues, and to further facilitate the navigation of the tree forest, we have created two additional encodings for the merger trees. The first is a summarization overview through the pixel heatmap described earlier. The second encoding is a horizontally aligned graph representation of the tree data, in which halos are represented by nodes, and merging or splitting operations are represented by arcs.

### 4.4. Immersion, 2D/3D Hybridization, and Interaction

The CAVE 2 environment is used in immersive mode for the 3D part of the application. Please note that in several figures used in this paper, stereo was turned off for better photo quality. We note that the CAVE2 environment has, like most second-generation, modern immersive theaters, no ceiling, or floor projection. This is due partly to display-technology constraints: while providing remarkably higher resolution and brighter environments than older systems with projected light, displays cannot provide passive/active stereo for multiple users at ceiling or floor locations. The other part of the rationale is due to collaboration requirements: for collaborative use, analysts wish to roll in desks and chairs to create a comfortable-enough environment; in this way, in addition to the environment immersive capabilities, they can continue to use their laptops and web-based technologies as they do in the office. When the environment is used for visual analytics activities, the room is also, generally, well lit. In our experience, light allows analysts to perceive better the facial and body cues of each other, as well as to take and consult notes, resulting in richer and more nuanced inter-disciplinary analyses.

**Figure 6:**
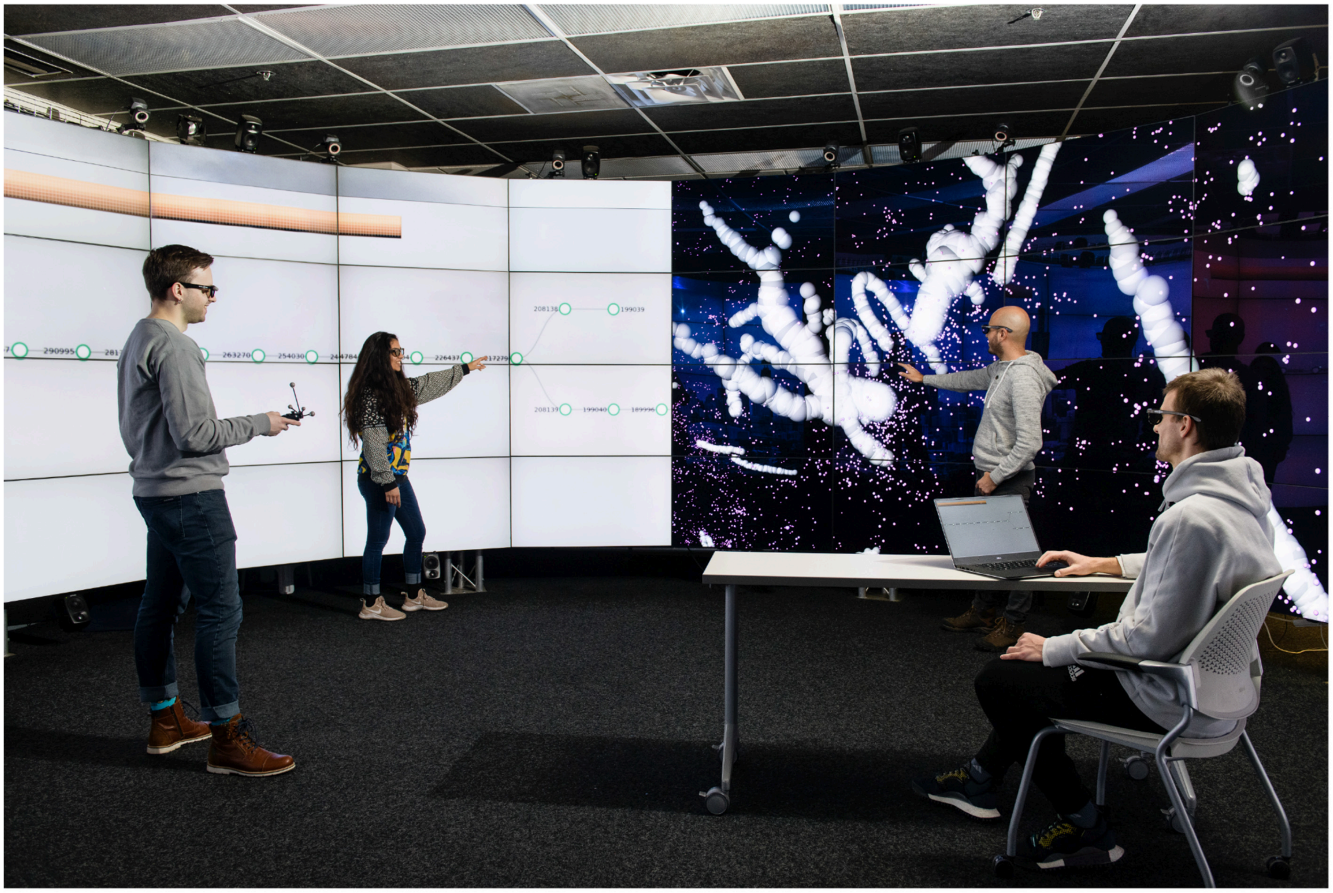
Hybrid usage of the CAVE2 environment, here shown in a 50/50 split between 2D and immersive 3D. Two researchers (left and right, standing) are referencing the immersive 3D structures in the right half of the CAVE2 environment, observed here in a timelapse. A third researcher points in the 2D representations in the left half of the CAVE2 the exact timestep where two major tree-structures merge. A fourth researcher, seated, interacts with the 2D representations using a laptop. In our experience, light allows analysts to perceive better the facial and body cues of each other, as well as to take and consult notes, resulting in richer and more nuanced inter-disciplinary analyses. In addition to the environment immersive capabilities, analysts can continue to use their laptops and web-based technologies as they do in the office.

For several demonstrations and for routine domain expert use, the CAVE2 environment was further split into 3D and 2D sections, taking advantage of the hybrid capabilities of the environment (Figure 6). The split ratio can be configured when starting the application. It is possible to have a complete 3D view and remove the 2D view, as shown in several figures in this paper. It is also possible to have a 50% split between 3D and 2D, as we frequently did for domain expert use, a 60/40 split and so on. To our knowledge, this is only the second known instantiation of such a hybrid use, after the brief, less documented NASA study referenced in our Related Work section.

The 2D views are displayed in the environment through the SAGE2 middleware, as a Chrome browser window. These window(s) are shown in the area of the environment that is used for 2D analysis, and can be easily controlled through a laptop. The advantage of using browser integration, as opposed to projecting the 2D views within the 3D immersive area, is that in this manner analysts can continue to easily access their typical web-based desktop workflow (web-browsing, email, spreadsheets, code etc.) within the environment. The 2D nonspatial representations are linked to the immersive spatial representations via the SAGE2 library and port communication.

In terms of interaction, one analyst drives the 3D part of the application using a wand and a tracked set of 3D glasses, while others analysts may observe the 3D results through 3D glasses and stereopsis. The 3D scene is rendered to the perspective of the tracked glasses, allowing that user to be fully immersed. Other users can experience immersion through the 3D glasses, although they experience the perspective of the user wearing the tracked glasses. The 3D glasses can be used while looking at the 2D view. The wand is tracked allowing for intuitive interaction, e.g., moving the wand in the up direction raises the scene.

A laptop was used for interacting with the 2D trees through SAGE2. The easy and intuitive interaction with a mouse on a laptop is an additional advantage of the browser encoding. The same or a different user can control/interact with the 2D view. The 2D view can also be observed by all analysts within the environment.

## 5. Evaluation and Results

Following the ACD theoretical model, we use a quantitative and qualitative methodology to evaluate our application. As noted by the US Department of Health and Human Services (Services, 2018), while references to evaluation methods frequently include the phrase “qualitative and quantitative methods,” “as if the mention of one method demands the inclusion of the other,” it remains “necessary to consider carefully the approach most suitable for answering the questions at hand, rather than reflexively calling for both” (Patton, 2002). Depending on the research questions to be answered, the best approach may be quantitative, qualitative, or some combination of the two. With this observation in mind, we designed our evaluation scheme along the research questions and the requirements behind this project.

Because the activity-centered paradigm emphasizes “why” and “how” questions over “how much/many” questions, we use a qualitative scheme to evaluate the impact of our application on the user workflow. Qualitative inquiry places a priority on people's lived experience and the meanings they ascribe to their experiences (Miles et al., 1994). As in this work, qualitative data often are collected in the settings under study, and they aim for rich description of complex ideas or processes, albeit typically across a limited number of individuals or settings. This approach stands in contrast to quantitative methods, which explore variables that can be captured or represented in numerical form, often across large samples and/or multiple points in time. In our case, the choice of a qualitative scheme was furthermore strongly supported by the following factors (Patton, 2002): (1) the nature of the halos project, which emphasizes exploring a new area of inquiry and generating hypotheses, without established measurements or known facts; (2) the general lack of a large sample of astronomy domain experts; (3) the general goal of generating information about how people involved with this project understand, think about, and make sense of the data. Conversely, these are equally strong arguments against a quantitative evaluation. As such, we quantitatively evaluate our application strictly with respect to its ability to serve the user activities.

### 5.1. Quantitative Evaluation

Using an 11 GB dataset, we were able to load the 89 timesteps for the particle data, and 89 timesteps for halos (starting at timestep 12, since the first 12 timesteps were very similar). The dataset includes 2M particle points per timestep in addition to the halo tree data; the number of trees ranges from 63 to 7 K. Loading the halo data takes about one minute, including the startup for the OmegaLib application. The particle data requires a negligible 400–550 ms longer to load than the halo data, due to the size of the dataset.

Our application attains a reasonable rate of 60 fps. Running intensive tasks, such as the time lapse function where multiple timesteps can be overlapped, can lead to a drop down to 20–30 fps. If we toggle the raw particle dataset off, and leave the halo dataset on, the application attains again 60 fps, even when every timestep is turned on.

### 5.2. Qualitative Evaluation

Qualitative research methods can play a powerful role in project evaluation, although they frequently are misunderstood and incompletely documented, giving rise to the idea that they are just not as rigorous and credible as quantitative methods. That is not true, although qualitative methods are less standardized than are quantitative methods. In this work, we used established qualitative methodology, including thoughtful and consistent data collection and analytic procedures.

Sample size in qualitative research is not judged by the same criteria as it is in quantitative research because statistical power is not the goal (Patton, 2002). Because this project explores a narrow phenomenon in depth (an analyst's process of making sense of halo data), we evaluated the application with two senior domain experts from the Adler Planetarium, who share key characteristics in terms of expertise: they both have significant experience in immersive environments and in astronomy data. Additionally, the project was demonstrated to several groups of visitors with varying levels of expertise.

One of the two experts is a co-author to this manuscript, and worked alongside the visualization researchers on the design, development, and iterative improvement of the application. This expert may thus exhibit response bias. The second expert only participated in the qualitative evaluation. The two Adler experts together evaluated the finalized prototype. In accordance with the activity-centered paradigm, no personal data was collected from the experts or from the groups of visitors. We made sure the experts were clear on the intentions of the research, and understood the evaluation was about the performance of the instrument/application and its support for their analysis workflow. The quotes reported in the manuscript were obtained using the think-aloud technique, qualitative observation, and semi-structured debriefing interviews. Furthermore, we used strategic and focused note taking both during the observation and the interviews. No audio or video recordings were made of the domain experts' interaction with the system.

With respect to the particle set and halo visualization, the experts found the visual encodings were effective. The interaction and flow were found to be “very smooth.” The immersive 3D representations were particularly appreciated for their ability to make clear spatial relationships among the halos. Furthermore, the experts remarked the density and distribution of particles inside halos “showed well the power of the halo.” The experts further appreciated the ability to analyze the relationship between mass and size by turning the particles off, since “halos could be very compact and still have high mass.”

One of the experts was particularly interested in merging halos, and found the time lapse visualization useful in that respect. The expert noted with interest that some halos evolve in parallel, never merge, and dissipate, while others merge into larger and larger structures, and wondered about the simulation conditions that made that behavior possible. The expert was surprised the simulation did not include temperature data, and concluded this was a cold dark matter simulation. Because this application was the first attempt to analyze halo data using a sophisticated visual analysis approach, there were no existing visualization solutions to compare against, and there were no known answers. Neither of the two major expert insights (cold dark matter and halo dissipation) were known facts to the expert prior to interacting with this application.

With respect to the merger trees, the experts expressed preference for the 2D, time-aligned representation of the tree, as opposed to the time lapse alternative encoding which traced the tree paths in 3D space. This was an interesting observation, attributable to the experts' familiarity with abstract representations and their interest in tree statistics. The domain experts still started their workflow with the details-first view of the particle and halo data, sifting for an interesting spatial pattern or timestep. The domain experts frequently analyzed the data in a hybrid 50/50 split between the 2D and the immersive representations, with repeated use of their laptops for quick web-based searches and spreadsheet analysis. The visualization researchers used their own laptops to repeatedly recode parts of the visual encodings.

The domain experts appreciated in particular the ability to examine details within context—without getting lost, despite the scale of the data. The experts also brainstormed for ways to port the application to the Adler museum, where OmegaLib was already installed and a 3D display is in use. In terms of for-improvement suggestions, the experts wished for a more varied range of colormaps for the data. A second suggestion was to include a stronger indication of the start and end of a timelapse, so the direction of the tree formation would be clear even without animating the sequence.

Last but not least in terms of domain expert usage, this scientific visualization application was recently installed in the CyberCANOE system at the Imiloa Planetarium (Hilo, Big Island of Hawaii), where it is used in astronomy talks. CyberCANOE is a stereoscopic wall system with three large 3D displays and a Kinect for tracking, and also handles hybrid 2D/3D displaying. Furthermore, the interdisciplinary committee behind the original visualization challenge and the Dark Sky dataset praised this project for its “stunning visualizations” and stated that “the domain scientists were all very impressed by the graphics” and “expressed strong interest in standing in front of the large display to describe their work to an audience.”

With respect to qualitative evaluation of group analysis, the Halo project is one of the most popular demonstrations in our lab. So far, the project has been shown to several groups of visitors, ranging from 6 to 45 people at one time. No personal data and no recordings were made during these demonstrations. We observed that the large immersive environment allows visitor subgroups to analyze together the data when a particularly interesting observation was made. The fact that some halos evolve in parallel, never merge, and dissipate is often noted and interpreted by visitors, as well as the emerging structures in the first timesteps of the simulation.

Navigation in the virtual environment comes natural to most visitors. We further noticed that analysts never lost track of the context of the data they were examining, despite the large scale of the data and their initial unfamiliarity with it. In fact several analysts were able to navigate toward an interesting area, and then do a precise 180° turn, and return to their previous location. Invariably and understandably, lay users tended to make less use of the hybrid non-spatial representations, which are of more interest and relevance to experts in the astronomy domain.

## 6. Discussion and Conclusion

Our results and evaluation show that the immersive halo visual analytics approach is useful and effective with respect to the functional specifications of the project, despite the large scale of the data. The approach integrates smoothly spatial and nonspatial encodings to enable data exploration. Immersive visualization further enabled analysts to navigate smoothly these large datasets and to detect spatial relationships among the halo structures.

In terms of scalability and portability, our approach benefits from the computing hardware behind the CAVE2 environment. However, we did not attempt datasets larger than the sample 11 GB dataset. Similarly, we did not explicitly tackle the multi-dimensional challenges of the multi-variate parameters associated with each halo. In terms of the generalizability and further quantification of our findings, we note that a formal user study would be interesting, although beyond the scope of this paper. Any such study should take particular care in the participant and task selection, given the central domain-knowledge aspects of the halo analysis problem, and the limited availability of domain experts.

Two of the most novel aspects of this immersive analytics application are its activity-focus and its details-first design. As also noted by Chen et al. (2016) and Luciani et al. (2019) were the first to remark, in 2016, that in “many scenarios, we often observe that an experienced viewer may find (overview first and details on demand) frustrating, as the viewer knows exactly where the interesting part of a detailed representation is.” Chen's observation is reflected in a vast number of works in scientific visualization that support explicitly spatial feature exploration first, and display the rest of the information primarily for context (e.g., Doleisch et al., 2003; Zhang et al., 2003; Hauser, 2005; Sherbondy et al., 2005; Caban et al., 2007; Jianu et al., 2009; Potter et al., 2009; Kehrer and Hauser, 2013). The “Details-first, show context, overview last” model (Luciani et al., 2019) we adopted in this work specifically applies to visual analytics processes where the features drive both the relevant context for the exploration process and the calculation of summarization overviews.

The third significantly novel aspect of this work is its usage of a hybrid environment, rarely documented in the immersive literature. We note the complementary benefits of our spatial and nonspatial encodings in the case of expert-level analysts, and conclude that Immersive Analytics require enough screen real estate to show multiple representations simultaneously. This types of Analytics also requires enough resolution to show context plus detail. Our environment further features the ability to show 3D everywhere, for all analysts in the room, even if 3D is not needed all of the time for analysis.

In terms of wider-scope lessons learned, we note first that this project was problem-driven and use-based, with the main hypothesis being that immersive visualization can help astronomers explore and analyze experimental data beyond the state of the art capabilities of computational analysis. Our evaluation indicates astronomers can indeed use immersive visualization to successfully analyze such data. In fact, both domain experts and lay users were able, through immersive visualization, to acquire new insights into the data. The immersion aspect of the visualization and navigation was furthermore important with respect to detecting and interpreting spatial relationships among spatially-distributed halo 3D structures.

Second, we note that high resolution immersive environments like the CAVE2 system have also limitations, in that they require domain experts to travel away from their office and potentially disrupt the experts' desk or lab-based workflow. Compared to our experience working with domain experts within the earlier, projection-based CAVE systems, we note the considerable willingness of our domain experts to travel to, and use repeatedly, the higher-resolution CAVE2 environment. The lesson emerging from this collaboration is that beyond higher-resolution capabilities, it is important to provide easy integration with the domain expert's typically web-based workflow, such as access to their laptop, notepad, spreadsheets, email, and web applications. As shown in Figure 6, the CAVE2 environment's design, which provides adequate lighting and space for rolling in desks and chairs, as well as integration with SAGE2, made possible this type of workflow integration. In terms of hybrid 2D/3D visualization, we note that 2D representations had value in detailed, expert-level analyses, and no significant value in the case of lay-audiences.

The last but not least major lesson emerging from this project is that adopting an activity-centered design paradigm (including an activity-based details-first approach), as opposed to focusing on an individual person, enabled us to create an immersive application that serves the needs of a wider community than the original expert user (our co-author). As hypothesized by Don Norman (The Design of Everyday Things, 3rd edition), “since people's activities around the world tend to be similar, and because people are quite willing to learn things that appear to be essential to the activity, activity should be allowed to define the product and its structure” (Norman, 2013). Our experience provides evidence in support of this hypothesis. As shown in our evaluation, our activity-driven project sparked further interest at the Adler Planetarium (Chicago, IL), and was adopted by a different group of astronomers at the Imiloa Planetarium (Hilo, Big Island of Hawaii)—researchers who have similar activities, although are different individuals. These results demonstrate the value of an activity-oriented approach to immersive visualization.

In conclusion, in this paper we described the design and evaluation of an immersive analytics tool for the exploration of large scale cosmological simulation data. Our design is based on a first instantiation in an immersive environment of the activity-centered theoretical paradigm, which emphasizes the intended use of the resulting visual analytics software, and demonstrates the success of this approach. Our design is also a first-documented successful instantiation in an immersive environment of the theoretical details-first visualization design paradigm. Our approach furthermore takes advantage of a state of the art hybrid immersive environment by integrating spatial and nonspatial data, while supporting implicit whole body navigation in this large space. The resulting application demonstrates that hybrid 2D and 3D immersive environments can augment the humans' ability to analyze and make sense of large and multifaceted datasets, while the resulting powerful astronomy application demonstrates the potential of activity-centered and details-first design to immersive visual analytics.

## Data Availability

The datasets analyzed for this study can be found in the Dark Sky Stanford University repository [http://darksky.slac.stanford.edu/scivis2015/].

## Author Contributions

GEM conceived, designed, directed, and lead the evaluation of this project. PH and KP implemented this project. JA provided essential feedback during the design process and assisted in the evaluation of the project. All authors contributed to the writing of the manuscript.

### Conflict of Interest Statement

The authors declare that the research was conducted in the absence of any commercial or financial relationships that could be construed as a potential conflict of interest.
